# Improvements to the HITS-CLIP protocol eliminate widespread mispriming artifacts

**DOI:** 10.1186/s12864-016-2675-5

**Published:** 2016-05-05

**Authors:** Austin E. Gillen, Tomomi M. Yamamoto, Enos Kline, Jay R. Hesselberth, Peter Kabos

**Affiliations:** Department of Medicine, University of Colorado Denver, Aurora, CO USA; Department of Biochemistry and Molecular Genetics, University of Colorado Denver, Aurora, CO USA; Present Address: Department of Bioengineering, University of Washington, Seattle, WA USA

**Keywords:** microRNA, HITS-CLIP, CLIP-seq, iCLIP, PAR-CLIP

## Abstract

**Background:**

High-throughput sequencing of RNA isolated by crosslinking immunoprecipitation (HITS-CLIP) allows for high resolution, genome-wide mapping of RNA-binding proteins. This methodology is frequently used to validate predicted targets of microRNA binding, as well as direct targets of other RNA-binding proteins. Hence, the accuracy and sensitivity of binding site identification is critical.

**Results:**

We found that substantial mispriming during reverse transcription results in the overrepresentation of sequences complementary to the primer used for reverse transcription. Up to 45 % of peaks in publicly available HITS-CLIP libraries are attributable to this mispriming artifact, and the majority of libraries have detectable levels of mispriming. We also found that standard techniques for validating microRNA-target interactions fail to differentiate between artifactual peaks and physiologically relevant peaks.

**Conclusions:**

Here, we present a modification to the HITS-CLIP protocol that effectively eliminates this artifact and improves the sensitivity and complexity of resulting libraries.

**Electronic supplementary material:**

The online version of this article (doi:10.1186/s12864-016-2675-5) contains supplementary material, which is available to authorized users.

## Background

The advent of HITS-CLIP and its derivative techniques has facilitated the mapping of RNA-protein interactions genome-wide in a variety of tissues and organisms [1, 2]. These techniques have made important contributions to the understanding of the function of a range of RNA-binding proteins, from the microRNA-related argonaute (AGO) to other regulatory factors, including FUS and PTBPs [3–5]. However, while useful, these techniques suffer from substantial mispriming during reverse transcription that can contaminate the resulting libraries with false peaks that may be difficult to distinguish from physiologically relevant peaks.

HITS-CLIP library construction [1] begins with the cross-linking of RNA-binding proteins to RNA using UV light, partial RNA digestion with RNAse A, and immunoprecipitation of the RNA-binding protein of interest and attached RNAs. The protein-bound RNAs are then dephosphorylated and a 3′ RNA adaptor is ligated with T4 RNA ligase. This step is performed “on-bead”, and the efficiency is highly dependent on the structure of the RNA-adaptor interaction [6]. The RNAs are then treated with PNK, again “on-bead”, and the protein-RNA complexes are resolved on an SDS-page gel to select only complexes of the expected size. The resulting size-selected protein-RNA complexes are then digested with Proteinase K to remove the protein and the remaining RNA is purified by phenol extraction and ethanol precipitation. A 5′ RNA linker is then ligated to the purified RNAs, and the RNA is reverse-transcribed using a primer perfectly complementary to the 3′ adaptor.

The use of a perfectly complementary reverse transcription primer results in substantial mispriming to suboptimal matches of the 5′ end of 3′ adaptor sequence in alternative RNAs during reverse transcription. Such mispriming events produce cDNAs from RNAs that may lack 3′ adaptor sequences. These cDNAs, which will be included in final sequencing libraries if they received a 5′ adaptor, contaminate the library with read pileups anchored to genomic occurrences of the adaptor sequence, rather than bona fide sites of protein binding. Recovery of irrelevant misprimed sequences is exacerbated by the fact that proteinase K digestion leaves one or two amino acids crosslinked to the RNA [7]. These crosslinked bases are frequent sites of termination during reverse transcription, meaning that only a fraction of reverse transcription events on crosslinked RNAs will read through to the 5′ adaptor and produce PCR-competent templates. This frequent early termination on crosslinked templates results in a bias towards RNAs that do not have crosslinking sites. This bias leads to overrepresentation of sequences not bound by the RNA-binding protein in the final library. Together, these problems introduce significant artifacts that make downstream analysis challenging.

HITS-CLIP does not provide direct insight into the microRNA (miRNA) involved in a given AGO-mRNA interaction, and the identification of putative miRNA:mRNA targets relies on *in silico* modeling of miRNA-mRNA interactions. Thus high levels of artifactual peaks would reduce the sensitivity of these computational approaches, possibly identifying false positive mRNA:miRNA pairings. Indeed, we show that widely accepted “wet” validation techniques that rely on overexpression of candidate miRNAs can confirm artifactual miRNA:mRNA interactions gleaned from HITS-CLIP. Here, we summarize our experiences with HITS-CLIP and propose effective experimental changes and bioinformatic steps to improve the construction and analysis of HITS-CLIP libraries. These improvements yield high confidence peaks that provide better insight into the biological system under study.

## Results

### Contamination of HITS-CLIP libraries with mispriming artifacts due to inefficient ligation

**Fig. 1:**
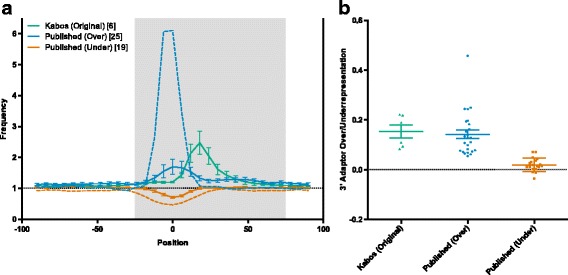
Mispriming on genomic occurrences of the 3′ adaptor sequence produces an artifact in HITS-CLIP data. **a** Occurrences of the first six bases of the 3′ adaptor (allowing for one mismatch) in 200 bp windows around peak centers plotted using 20 bp sliding windows (with a 6 bp shift between each window) relative to the expected frequency of each adaptor-complement (calculated using 1 x 10^6^ randomly sampled exonic sequences of 200 bp). Our early samples (emerald; 6 samples from 1 research group) show consistent overrepresentation of the adaptor sequence. This overrepresentation is also seen in a group of published samples (blue; 25 samples from 10 groups), while another group of published samples show underrepresentation of the adaptor sequence at the center of the peak (vermilion; 19 samples from 9 groups). The samples with the most extreme over- and underrepresentation are shown as dashed blue and vermilion lines, respectively. **b** Percentage of peaks containing the first six bases of the 3′ adaptor sequence (allowing for one mismatch) between positions −25 and +75 in each peak (highlighted in grey in A), minus the expected frequency (calculated using 1 x 10^6^ randomly sampled exonic sequences of 200 bp). Groups are the same as in (**a**)

We observed substantial overrepresentation of 3′ adaptor sequence due to mispriming on genomic sequence in our early HITS-CLIP samples (Fig. 1a, emerald line). In the most severely affected regions of these samples, the first six bases of the 3′ adaptor (allowing a one base mismatch) are observed at a frequency more than 2-fold higher than expected from randomly sampled exonic sequences. To determine if this problem also exists in published HITS-CLIP datasets, we analyzed peak calls for all HITS-CLIP experiments available in starBase 2.0, a collection of published CLIP-seq data curated and hosted by the RNA Information Center, State Key Laboratory for Biocontrol, Sun Yat-sen University, Guangzhou, China [8]. We analyzed peak calls for 44 HITS-CLIP experiments from 17 research groups, including 15 target proteins and 6 different 3′ adaptor sequences in both human and murine cells and tissues [4, 5, 9–29]. We found that the experiments segregated into two groups, based on the presence of the mispriming artifact (Fig. 1a). The first group (Fig. 1a, blue line), consisting of 25 samples from 10 research groups, shows pronounced overrepresentation of the adaptor sequence at the center of the peak and 3′ of the center of the peak. The position of the overrepresentation is dependent on the peak calling algorithm used in each publication, and is thus more variable than our data shown in emerald. These samples demonstrate that mispriming is a widespread problem in published HITS-CLIP datasets, with an average of over 1.5 times the expected presence of the adaptor sequence at the center of the peak, and a maximum observed frequency of more than 6-fold greater than expected (dashed blue line). The second group (Fig. 1a, vermilion line) consists of 19 samples from nine groups, and shows a distinct underrepresentation of the adaptor sequence at the center of the peak. This suggests that either these peaks have been filtered to remove artifactual peaks resulting from mispriming, that we have not correctly identified the adaptor sequence used in library preparation, or that these libraries do not contain substantial mispriming artifacts. We confirmed the adaptor sequence for each sample by examining the FASTQ files for sequenced adaptor dimers, making it unlikely that representation of the incorrect adaptor sequence is being assessed. We also re-analyzed these data using our analysis pipeline, and found that the majority of the published peak sets are complete representations of the publicly available raw data, with no evidence of bioinformatic filtering (data not shown). Thus, while it is possible that a small number of published peak calls may have been bioinformatically filtered, the majority of these samples lack substantial mispriming artifacts. As such, it is likely that underrepresentation of the 3′ adaptor sequence at the center of the peak is due to the overrepresentation of functional sequences (i.e., binding motifs) at this site. The lack of mispriming is likely due to differences in immunoprecipitation efficiency and RNA binding efficiency of the antibodies used and RNA binding proteins that were assayed.

Next, we attempted to estimate the number of artifactual peaks in each sample, based on the over- or underrepresentation of the 3′ adaptor sequence between positions −25 and +75 in each peak (this region, which shows pronounced overrepresentation of the adaptor sequence in affected samples, is highlighted in grey in Fig. 1a). We found that an average of 14.2 % of peaks in the overrepresentation group (Fig. 1a, blue line) are likely artifacts, with a low of 5.5 % and a high of 45.8 % (Fig. 1b, blue line). This is comparable to our samples (Fig. 1b, emerald line), in which 15.3 % of peaks are likely artifacts In contrast, the underrepresentation group (Fig. 1a, vermilion line) is effectively free of the artifact, with the 3′ adaptor sequence observed at a modest 2 % higher than expected (Fig. 1b). Finally, we attempted to see if any obvious factors could account for the variable presence of the artifact in the public datasets. We assessed the contribution of target species (human vs. mouse), target protein (AGO vs. non-AGO), RT conditions and the number of peaks per library, but none were significantly correlated with overrepresentation of the 3′ adaptor sequence (data not shown). We also considered the sequence of the RT primer. Only two reverse transcription primers were used in more than one publication in StarBase: the primer from the 2005 CLIP protocol [1] (5′-CCGCTGGAAGTGACTGACAC-3′) and the primer from the Illumina Small RNA Kits (5′-CAAGCAGAAGACGGCATACGA-3′). Each primer was used in 17 samples, and no significant difference in artifact representation was observed between the two primers (5.1 % vs. 5.2 % overrepresentation; Additional file 1: Figure S1). The four remaining primers were used by only one group each (1–3 samples each), making it impossible to separate the sequence of the primers from other factors affecting artifact representation. Finally, we considered the impact of the two major modifications to the HITS-CLIP protocol (iCLIP and PAR-CLIP) on mispriming during RT. We analyzed the 10 iCLIP datasets in StarBase 2.0 (10 samples from two research groups), and found only slight overrepresentation (25 %) of the complement of the reverse transcription primer on average (Additional file 2: Figure S2). While the artifact is much better controlled in the iCLIP libraries than in HITS-CLIP libraries, it is still present. PAR-CLIP, which uses photoreactive ribonucleoside analogs to map crosslinking sites, allows legitimately crosslinked sequences to be identified based on the presence of mutated bases. While mispriming may still occur in these datasets, non-crosslinked sequences (including products of mispriming) can be effectively filtered bioinformatically.

### A nested reverse transcription primer, combined with a protected reverse PCR primer, eliminates mispriming artifacts

**Fig. 2:**
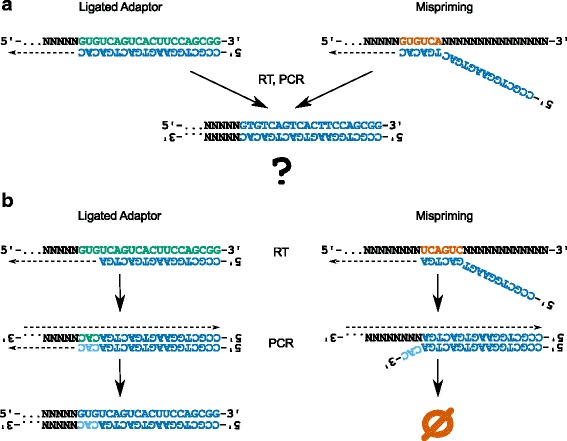
Identification of misprimed reads using a nested reverse transcription primer. **a** When the reverse transcription primer (blue) is flush with the 3′ adaptor, reads originating from 3′ adaptor (emerald) priming and mispriming (vermilion) are indistinguishable, as both are competent PCR templates. **b** However, when a nested reverse transcription primer (blue) is used along with a protected reverse PCR primer (bases with phosphorothioate bonds are shown in sky blue), only reads originating from 3′ adaptor (emerald) priming are valid templates for PCR amplification. Reverse transcription products derived from mispriming are not amplified in subsequent PCR steps, as they do not contain the final 3 bases of the reverse PCR primer. This 3-base mismatch prevents elongation of the primer by the polymerase, and the phosphorothioate bonds prevent the ‘chew-back’ of the primer by exonuclease activity of the polymerase

**Fig. 3:**
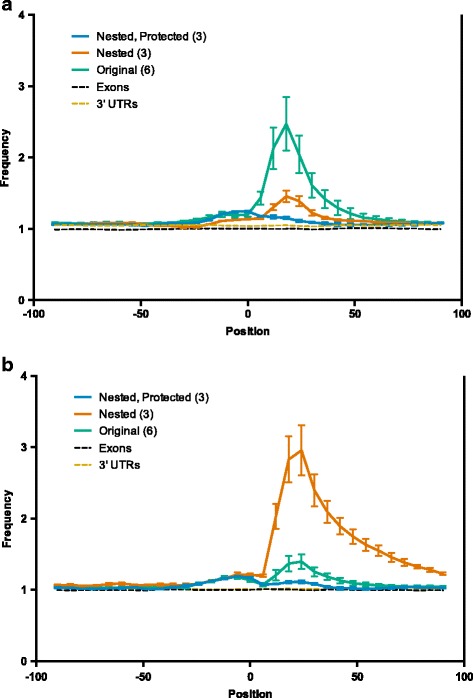
Removal of reads derived from mispriming events eliminates artifactual peaks. Occurrences of the first six bases of the 3′ adaptor (allowing for one mismatch) (**a**) or bases 4–9 of the 3′ adaptor (**b**) in 200 bp windows around peak centers plotted using 20 bp sliding windows (with a 6 bp shift between each window) relative to the expected frequency of each adaptor-complement (calculated using 1 x 10^6^ randomly sampled exonic sequences of 200 bp). When using the original HITS-CLIP protocol (emerald), significant mispriming is observed. The use of a nested RT primer reduces the overrepresentation of the adaptor sequence (vermilion, **a**), but results in overrepresentation of the sequence complementary to the 3′ end of the RT primer (vermilion, **b**). Finally, using a nested RT primer and protected PCR primer results in much more even representation of both the adaptor sequence (blue, **a**) and RT primer complement (blue, **b**), with only modest overrepresentation of each sequence around the center of the peak

In order to reduce the effect of mispriming events without fundamentally changing the technique, we examined the use of a nested reverse transcription primer (also used in the iCLIP protocol [30]) and the protection of the 3′ end of the reverse PCR primer from the 3′ to 5′ exonuclease activity of the polymerase (Fig. 2). By using a nested reverse transcription primer that lacks the first three bases of the 3′ adaptor, along with a full-length reverse PCR primer with phosphorothioate bonds protecting the final four bases, only cDNAs resulting from priming on 3′ adaptor sequences will be valid templates for PCR amplification. We used a nested primer with and without a 3′ protected reverse primer on three samples each. The results are shown in Fig. 3, with our original six samples included for reference (Fig. 3a, emerald line). The use of a nested primer (Fig. 3a, vermilion line) resulted in a substantial reduction in overrepresentation of the first six bases of the 3′ adaptor. However, this group exhibited significant overrepresentation of bases 4–9 of the 3′ adaptor, which are complementary to the final six bases of the nested reverse transcription primer (Fig. 3b, vermilion line). This result is consistent with mispriming during reverse transcription and polymerase-mediated 3′ to 5′ exonuclease activity on the unprotected 3′ end of the reverse PCR primer during PCR, essentially shifting the mispriming artifact 3 bases to match the 5′ end of the nested reverse transcription primer. This evidence of exonuclease activity highlights the importance of protecting the 3′ end of the reverse PCR primer when paired with a nested reverse transcription primer. The use of a nested primer with a 3′ protected reverse primer results in much more even representation of the adaptor sequence, with only modest overrepresentation around the center of the peak (Fig. 3, blue line). This modest overrepresentation is likely due to the enrichment of regions legitimately bound by Ago that contain this sequence. Crucially, when the protected PCR primer is used, bases 4–9 of the 3′ adaptor are not overrepresented (Fig. 3b, blue line), demonstrating that cDNAs derived from mispriming were not amplified in subsequent PCR steps. In samples shown in Fig. 3a-b (blue and vermillion lines), we also enzymatically digested unincorporated primer following reverse transcription using Exonuclease I. This treatment prevented the production of PCR products derived from the forward PCR primer combined with RT primer carried over from the first PCR. These products, which can use cDNA resulting from mispriming or legitimate adaptor priming as a template, deplete the forward primer pool but lack the reverse priming site for the second PCR. Thus, the amplification of these products reduces library complexity by limiting the production of valid templates for the second PCR. While this digestion had no effect on the presence of the mispriming artifact, it did modestly increase library complexity, with ExoI digestion resulting in 5.3 % more reads remaining after deduplication (*n* = 2).

### Removal of artifactual reads eliminates false peaks and improves sensitivity, but does not affect physiologically relevant peaks

**Fig. 4:**
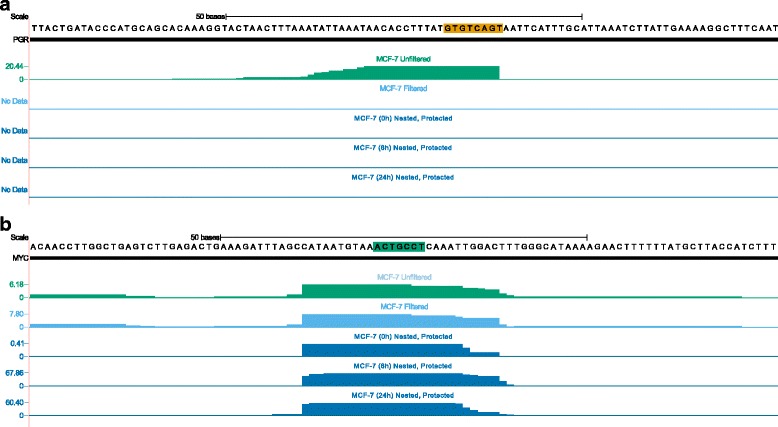
True peaks are indistinguishable from artifacts in most HITS-CLIP data. **a**. A CLIP peak in the Progesterone Receptor (PGR) 3′ UTR (emerald; original protocol) in MCF-7 cells is the result of mispriming on a perfect reverse-complementary match to the final 8-bases of the RT primer (highlighted in vermilion). This sequence is also complementary to miR-888, making the resulting peak appear to contain a miR-888 target. This peak disappears completely when the reads are filtered (sky blue) or a nested RT primer and protected PCR primer is used (blue; 0, 6 and 24 h after stimulation with estradiol). **b**. A robust CLIP peak in the c-Myc 3′ UTR is a bonafide target of miR-34b (seed complement highlighted in emerald) [32–35]. This peaks is also present when the data are filtered (sky blue) and when a nested RT primer and protected PCR primer are used (blue). Figure based on output from http://genome.ucsc.edu (hg19 assembly) [42, 43]

**Fig. 5:**
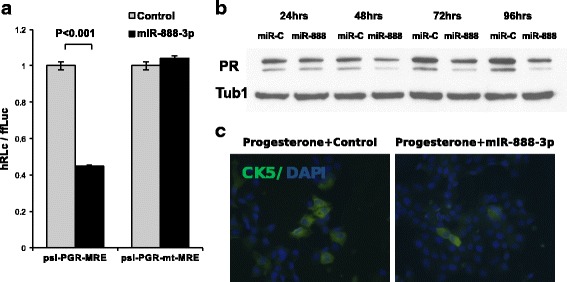
miR-888 overexpression represses Progesterone Receptor expression. **a**. Luciferase reporter assays confirm functional binding of overexpressed miR-888-3p to PGR-MRE and disruption of this interaction upon mutation the seed-binding region. **b**. Western blot analysis of BT474 cells transfected with miR-888 or control (5 nM each). MiR-888-3p overexpression represses PGR expression as compared to cells transfected with control mimic. **c** Progesterone treatment increases the number of CK5 expressing cells in ER+ breast cancer cells (T47D are shown). MiR-888-3p overexpression effectively blocks the progesterone induced increase of CK5

HITS-CLIP is often used to identify miRNA-target interactions, which are then validated using other experimental methods. These methods frequently consist of overexpression of the miRNA, followed by measurement of target mRNA/protein expression or 3′ UTR reporter assays. Figure 4 shows two peaks from HITS-CLIP performed on MCF-7 using the original “flush” RT primer. One, in the Progesterone Receptor (PGR) 3′ UTR, is the result of mispriming on a perfect reverse-complementary match to the final 8-bases of the RT primer (highlighted in vermilion). This sequence is also complementary to miR-888, making the resulting peak appear to contain a miR-888 target. The other peak, located in the c-Myc (MYC) 3′ UTR, is centered on a miR-34b seed site match (highlighted in emerald). Both peaks are similar in appearance in the top track (MCF-7, original primer, no filtering; emerald), but the PGR peak disappears completely when reads are filtered as described previously [31] (MCF-7, original primer, filtered; sky blue) or when a nested RT primer and protected PCR primer are used (MCF-7, 0, 6 and 24 h treatment with estradiol; blue). The c-Myc peak, in contrast, retains a consistent position and shape after removing artifactual reads. Not unexpectedly, the c-Myc-miR-34b interaction has been experimentally validated in several reports [32–35]. However, using standard miRNA overexpression-based validation techniques, the PGR peak also appears to demonstrate a valid interaction with miR-888 (Fig. 5). We first demonstrated that overexpression of miR-888-3p represses expression of a luciferase reporter carrying the putative target site in the 3′ UTR, and that mutation of this site completely relieves the repression (Fig. 5a). We also showed that endogenous PGR protein expression is reduced following overexpression of miR-888-3p, relative to a control miRNA (Fig. 5b). We were even able to demonstrate functional repression of PGR activity by miR-888, as overexpression of the miRNA prevented the upregulation of keratin 5, a known downstream target of PGR, in response to progesterone treatment (Fig. 5c). While this appears to demonstrate the robust, physiologically relevant regulation of PGR by miR-888 in MCF-7 cells, libraries prepared with a nested, protected reverse transcription primer reveal that miR-888 is not expressed at a measurable level in the MCF-7 cell line (data not shown).

**Fig. 6:**
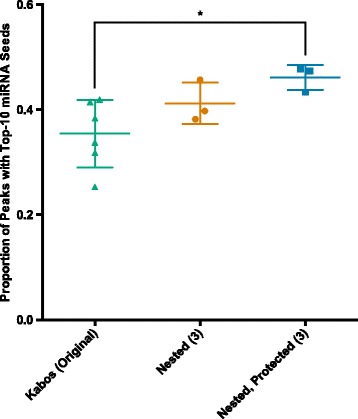
A nested RT primer and protected reverse PCR primer produce high confidence AGO HITS-CLIP peaks and increase sensitivity of interaction detection. Percentage of peaks containing the reverse complement of the 6 bp miRNA seed site for at least one the 10 most highly expressed miRNAs in that sample (measured in the same CLIP experiment). The use of a nested RT primer and protected PCR primer (Blue; *n* = 3) results in a significant, 30 % increase in the percentage of peaks, with a top-10 miRNA seed site relative to the original protocol (emerald; *n* = 6), while the use of a nested RT primer alone (vermillion; *n* = 3) produces a 16 % increase. *p ≤ 0.05

To assess the impact of our modifications on library quality, we considered genome-wide measurements of miRNA binding and the sensitivity of peak detection. First, we counted the number of peaks containing sequences complementary to the seed sites of the ten most highly expressed miRNAs in each sample. We found that the use of a nested reverse transcription primer and protected reverse PCR primer results in a statistically significant (*p* = 0.03) 30 % increase in the proportion of peaks containing seed matches for the ten most highly expressed miRNAs (Fig. 6a). A modest improvement was also observed with the use of a nested primer alone (Fig. 6a, blue circles), but the increase was not significant. These data demonstrate that our modifications improve the accuracy of AGO2 binding maps generated using HITS-CLIP and provide better insight into the biological system under study than the unmodified protocol. Next, we considered the impact of library contamination on the sensitivity of peak detection in our samples. For each of three matched pairs of libraries generated with a nested RT primer (containing mispriming artifacts) or a nested RT primer and a protected reverse PCR primer (no mispriming artifacts), we measured the number of peaks called. To normalize for library size, we conservatively called peaks on three sets of four million randomly sampled alignments from each library, and compared the average number of significant peaks called for each paired sample. We found that the libraries prepared with the nested, protected primers contained an average of 83 % (SEM = 19.1 %) more statistically significant peaks of Ago enrichment per alignment than the paired libraries constructed with a nested RT primer alone. This increased sensitivity allows for the detection of weaker Ago2-mRNA interactions, substantially improving library quality.

## Discussion

We found that the majority of published HITS-CLIP libraries either contain substantial mispriming artifacts or appear to have been processed to remove these artifacts, and that these artifacts can lead to false positive peaks that pass standard validation techniques. The mispriming in the most severely affected libraries may simply be a symptom of generally poor library quality, but without proper controls (libraries prepared in parallel with and without our modifications), it is impossible to differentiate between mispriming resulting from poor library quality and mispriming causing poor library quality. Whatever the cause, this issue is underappreciated, as none of the publications represented in StarBase make any mention of this pervasive artifact, or of any explicit steps taken to mitigate the artifact. It is important to recognize and address this problem, as HITS-CLIP is routinely used to provide evidence of miRNA-mRNA interactions under physiological conditions, in addition to many other RNA-protein interactions. Without such evidence, it is difficult to assess the validity of reported miRNA-mRNA interactions, as most commonly accepted validation techniques (reporter assays, target transcript and protein quantification, etc.) merely provide evidence for binding capacity rather than biologically relevant interaction. As we have demonstrated, validation techniques can yield false positive results when supported by artifactual HITS-CLIP data. We show that an artifactual HITS-CLIP peak (Fig. 4) leads to “validation” of a robust interaction between (overexpressed) miR-888 and endogenous Progesterone Receptor in MCF-7 cells (Fig. 5), including repression of downstream targets of PGR (CK5; Fig. 5c). Overexpressing miR-888 represses PGR expression, but this is not surprising, as the miRNA is highly complementary to the target. While miR-888 is clearly capable of regulating PGR expression, and may do so in other cell types, there is no evidence that it does under normal conditions in MCF-7, where it is not expressed at detectable levels. Here, we present a modification that mitigates the problem while making minimal changes to the original HITS-CLIP protocol. In the case of AGO HITS-CLIP, our modifications to the method and downstream analysis result in high confidence measurements of the miRNA-mRNA interactome under physiologic conditions. These measurements provide direct support for miRNA-target interactions inferred by other methods, and provide biological relevance to downstream functional assays.

There are two existing variants of HITS-CLIP that partially address the problems described here: PAR-CLIP and iCLIP [30, 36]. PAR-CLIP (Photoactivatable-Ribonucleoside-Enhanced Crosslinking and Immunoprecipitation) differs from HITS-CLIP in that it uses the incorporation of photoreactive ribonucleoside analogs, such as 4-thiouridine (4-SU) and 6-thioguanosine (6-SG), to map sites of crosslinking. Cells are grown in the presence of these analogs, which are incorporated into nascent RNA transcripts. The use of these ribonucleoside analogs results in both improved RNA recovery and the introduction of a marker of crosslinking, in the form of mutations at the site of crosslinking, that is detectable in sequencing. While PAR-CLIP is subject to the same potential for mispriming as HITS-CLIP, excluding reads without mutations introduced by crosslinking can be used to effectively retain only legitimately crosslinked sequences. This will eliminate the majority of misprimed sequences, which are not crosslinked. However, while non-crosslinked sequences can be removed from PAR-CLIP libraries, the presence of substantial mispriming artifacts will still reduce effective library size and library quality. Thus, the same nested RT primer approach suggested here for HITS-CLIP can be applied to PAR-CLIP, with similar benefits.

iCLIP (individual-nucleotide resolution Cross-Linking and Immunoprecipitation) is much more effective than either unmodified HITS-CLIP or PAR-CLIP at mitigating the artifact described here because it addresses the issue of reverse transcription termination at sites of crosslinking. This is accomplished by reverse transcribing with a primer complementary to the 3′ adaptor that also includes the 5′ adaptor sequence. The resulting cDNAs are then circularized and cut at a site within the RT primer to produce a linear cDNA with 5′ and 3′ adaptor sequences. Unlike HITS-CLIP and PAR-CLIP, the resulting cDNAs are PCR-competent regardless of where reverse transcription terminated, removing the bias introduced by requiring the reverse transcriptase to read through the crosslinking site to the ligated 5′ adaptor. This increased efficiency, along with the use of a nested RT primer, likely explains the lower rate of mispriming artifacts observed in iCLIP libraries (Additional file 2: Figure S2). Termination at the site of crosslinking also allows for the identification of crosslinking sites indirectly through the mapping of 5′ ends of cDNAs (reverse transcription termination sites). Despite the fact that the mispriming artifact is present at low levels in the iCLIP libraries we analyzed (Additional file 2: Figure S2), there has been at least one attempt to mitigate the inefficiency of RNA ligation in iCLIP, though no explicit rationale is given for the change in protocol [37]. In this case, the authors used a biotinylated 3′ adaptor, which allowed for enrichment of RNA-protein complexes with ligated adaptors using an avidin pull-down. While this should improve the efficiency of reverse transcription on legitimate templates by enriching for adaptor-ligated RNAs, this represents a substantial alteration to the iCLIP protocol, and has the undesirable effect of adding another pull-down to an already complex protocol while still not taking full advantage of the nested iCLIP reverse transcription primer. A much more satisfying solution, either alone or in combination with the biotinlyated 3′ adaptor, is to use a 3′ protected reverse PCR primer, as we have described here for HITS-CLIP. No matter which approach is taken to improve HITS-CLIP, it is important to recognize the limitations of the technique and process the data appropriately.

## Conclusions

Here, we describe the use of a nested reverse transcription primer and a protected reverse PCR primer in place of the original oligonucleotide primers to eliminate widespread mispriming artifacts in HITS-CLIP. These simple but effective modifications help to improve HITS-CLIP library quality by producing high-confidence peak calls that accurately represent the RNA binding protein-RNA interactome under physiological conditions. Specifically, removal of artifactual reads both eliminates false peaks (while not affecting physiologically relevant peaks) and improves sensitivity, allowing for the identification of weaker interactions. These modifications have the potential to improve library quality not just for AGO HITS-CLIP, as described here, but for all HITS-CLIP targets and all library construction techniques which rely on the ligation of a 3′ adaptor to RNA.

## Methods

### Artifact frequency

For each set of peak calls generated in our lab or obtained from StarBase v2.0 [8], we expanded the peaks to a uniform 200 bp (around the existing center of the peak). We then counted the number of occurrences of the first six bases of the 3′ adaptor (allowing for one mismatch) in the uniform peaks using 20 bp sliding windows (with a 6 bp shift between each window), and plotted this data as the fold-difference relative to the expected frequency of each adaptor-complement (using 1 million randomly sampled genomic sequences of 200 bp). We confirmed the 3′ adapter sequence in each library by downloading the raw data from NCBI SRA (www.ncbi.nlm.nih.gov/sra) and examining the reads directly and with FASTQC (http://www.bioinformatics.babraham.ac.uk/projects/fastqc/).

### HITS-CLIP

HITS-CLIP was performed as in [3 N], with the following modifications: A reverse transcription primer with the sequence 5′-CCGCTGGAAGTGACTGA-3′ was used in place of the “DP3” primer in the original method. Following reverse transcription, the reaction was treated with Exonuclease I (NEB) to remove unincorporated reverse transcription primer. Specifically, 10 U of ExoI was added to each sample and they were incubated at 37C for 30 min followed by heat inactivation of ExoI at 80C for 20 min. A 5′ extension was added to the reverse PCR primer to allow for multiplexing and use on the Illumina Hiseq platform, and phosphorothioate bonds were added between the final four bases to prevent cleavage by exonuclease activity of the polymerase (5′-GTGACTGGAGTTCAGACGTGTGCTCTTCCGATCCCGCTGGAAGTGACTGA*C*A*C-3′, “*” indicate phosphorothioate bonds). The second PCR was performed with index primers compatible with the new first-stage primers (5′-CAAGCAGAAGACGGCATACGAGATCGGT NNNNNN GTGACTGGAGTTCAGACGTGTGCTCTTCCGATC-3′).

### Computation analysis

Reads were processed with cutadapt (https://code.google.com/p/cutadapt/) [38] to remove 3′ adaptor sequences and 3′ bases with QUAL < 10. Trimmed reads were aligned to the genome using GSNAP (http://research-pub.gene.com/gmap/) [39, 40], and peaks were called on alignments with MAPQ >10 using piranha (http://smithlabresearch.org/software/piranha/). Overlapping peaks were then merged and all peaks were resized to a uniform 60 bp centered on the summit of each peak using peaktools (https://github.com/jayhesselberth/peaktools). To normalize library sizes for peak calling sensitivity assessment, 4 million aligned reads were randomly sampled without replacement from each aligned library by shuffling the alignment order with GNU shuf (seeded with/dev/random) and retaining the first 4 million alignments.

### Cell culture, transfection and treatment assays

The human breast cancer cell lines T47D and MCF7 were originally obtained from Iafa Keydar [9], and Sam Brooks, the Michigan Cancer Foundation, respectively. Cells were maintained in MEM supplemented with 5 % fetal bovine serum (FBS) and penicillin/streptomycin. BT474 cells were obtained from ATCC (Manassas, VA) and maintained in DMEM medium supplemented with 5 % FBS and penicillin/streptomycin. HEK-293 T cell line was obtained from ATCC (Manassas, VA) and maintained in DMEM with 10 % FBS. Cell line authenticity was confirmed by short tandem repeat analyses in the University of Colorado DNA Sequencing Core Laboratory [20].

### Plasmids, viral constructs and DNA cloning

Luciferase reporter vectors were created by cloning of the miRNA Responsive Element (MRE) sequences into the XhoI-NotI restriction site of psi-Check2 vector (Promega, Madison, WI) as previously reported [41]. Retroviral constructs for stable overexpression of miR-888 were constructed as previously reported [10].

### Luciferase assays

HEK-293 T cells were plated at approximately 40 % confluence (50,000 cells per well) in 24 well plates. Sixteen hours later the psi-Check2 vectors and miR mimic/control mimic were co-transfected using Lipofectamine 2000 as per manufacturer’s protocol. Cells were harvested by passive lysis and assayed for firefly and renilla luciferase activity using Dual Luciferase Reporter Assay kit on a Glomax luminometer (Promega) following manufacturer’s instructions. The student’s *t*-test was used to determine significance.

### Western blotting

Cell lysates were quantitated for protein content, and approximately 25 mg lysates were resolved by standard SDS-PAGE and transferred to PVDF membrane. Blots were probed by antibodies against PGR (Cell Signaling Technology, Danvers, MA) and an appropriate HRP-conjugated secondary antibody. Blots were then stripped and reprobed for alpha tubulin (Sigma-Aldrich, St. Louis, MO) to ensure equal protein loading. The blots were then visualized using enhanced chemoluminescence.

### Immunocytochemistry

Cells were fixed onto glass coverslips in ice-cold 70 % acetone/30 % methanol for 5 min and stained by immunocytochemistry (ICC) with antibodies to CK5 (Dako, Glostrup, Denmark). Secondary antibodies were anti-mouse and anti-rabbit Alexa Fluors 488 (green). Nuclei of cells were counterstained with DAPI. A Nikon E600 microscope (Nikon Corporation, Tokyo, Japan) was used for photography. Images were shot in black and white using ImagePro software (Media Cybernetics, Rockville, MD) and merged and pseudo-colored in Adobe Photoshop CS3 (Adobe Systems, San Jose, CA).

### Availability of supporting data

All publicly available HITS-CLIP and iCLIP data supporting the results of this article are available at StarBase 2.0 (http://starbase.sysu.edu.cn/).

The original HITS-CLIP datasets supporting the conclusions of this article are available in NCBI Sequence Read Archive (SRA) studies SRP042140 (http://trace.ncbi.nlm.nih.gov/Traces/sra/?study=SRP042140) and SRP070520 (http://trace.ncbi.nlm.nih.gov/Traces/sra/?study=SRP070520).

## Additional files

Additional file 1: Figure S1. The sequences of the two most common adaptor/primer pairs do not influence the frequency of mispriming. Percentage of peaks containing the first six bases of the 3′ adaptor sequence (allowing for one mismatch) between positions −25 and +75 in each peak (highlighted in grey in Fig. 1a), minus the expected frequency (calculated using 1 x 10^6^ randomly sampled exonic sequences of 200 bp). Adaptors/primers from [1] blue, *n* = 17) and the Illumina Small RNA Kit (vermillion, *n* = 17) are included. (PDF 19 kb) Additional file 2: Figure S2. Mispriming is also detectable in iCLIP libraries. Occurrences of the first six bases of the 3′ adaptor (allowing for one mismatch) in 200 bp windows around peak centers plotted using 20 bp sliding windows (with a 6 bp shift between each window) relative to the expected frequency of each adaptor-complement (calculated using 1 x 10^6^ randomly sampled exonic sequences of 200 bp). iCLIP samples (blue; 10 samples from 2 research groups) show consistent underrepresentation of the adapter sequence across the peaks, with mild overrepresentation immediately downstream of the center of the peak. The sample with the most extreme overrepresentation is shown as a dashed blue line. (PDF 10 kb)
