# Supplementary figures and images for: Improvements to the HITS-CLIP protocol eliminate widespread mispriming artifacts
Source: BMC Genomics. 2016 May 5;17:338. doi: 10.1186/s12864-016-2675-5 (PMC4858895; doi:10.1186/s12864-016-2675-5)

3' Adaptor Over/Underrepresentation

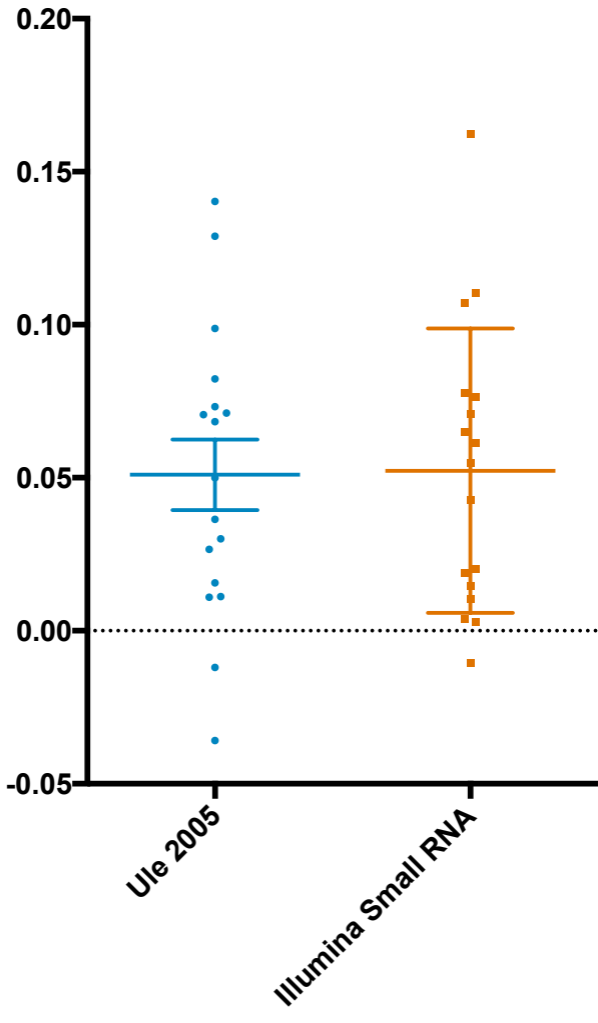

Supplement: Additional file 1: Figure S1. — The sequences of the two most common adaptor/primer pairs do not influence the frequency of mispriming. Percentage of peaks containing the first six bases of the 3′ adaptor sequence (allowing for one mismatch) between positions −25 and +75 in each peak (highlighted in grey in Fig. 1a), minus the expected frequency (calculated using 1 x 10^6^ randomly sampled exonic sequences of 200 bp). Adaptors/primers from [1] blue, n = 17) and the Illumina Small RNA Kit (vermillion, n = 17) are included. (PDF 19 kb) [file 12864_2016_2675_MOESM1_ESM.pdf]

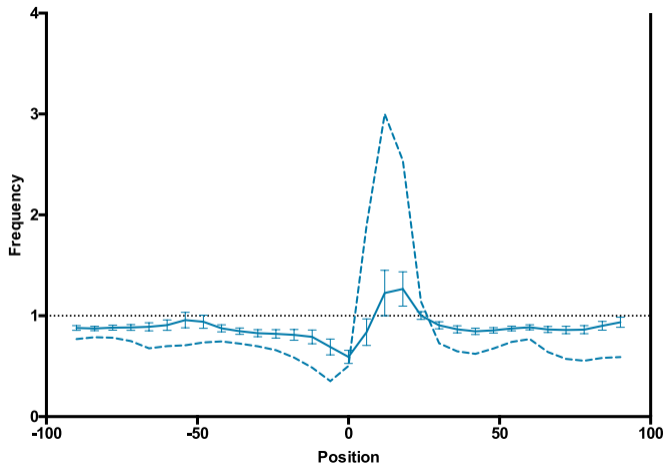

Supplement: Additional file 2: Figure S2. — Mispriming is also detectable in iCLIP libraries. Occurrences of the first six bases of the 3′ adaptor (allowing for one mismatch) in 200 bp windows around peak centers plotted using 20 bp sliding windows (with a 6 bp shift between each window) relative to the expected frequency of each adaptor-complement (calculated using 1 x 10^6^ randomly sampled exonic sequences of 200 bp). iCLIP samples (blue; 10 samples from 2 research groups) show consistent underrepresentation of the adapter sequence across the peaks, with mild overrepresentation immediately downstream of the center of the peak. The sample with the most extreme overrepresentation is shown as a dashed blue line. (PDF 10 kb) [file 12864_2016_2675_MOESM2_ESM.pdf]
